# Standardized Culture of Skin Fibroblasts From Punch Biopsies for Germline DNA Isolation in Myeloid Malignancies: A Practical Bedside-to-Laboratory Approach

**DOI:** 10.21769/BioProtoc.5469

**Published:** 2025-10-05

**Authors:** Parampreet Kour, Nedhi Kumari, Naveen Kaushal, Tanvi Sharma, Shano Naseem, Jogeshwar Binota, Manupdesh Singh Sachdeva, Arihant Jain, Manish Rohilla, Reena Das, Pankaj Malhotra, Pulkit Rastogi

**Affiliations:** 1 Department of Haematology, Postgraduate Institute of Medical Education and Research, Chandigarh, India; 2 Department of Biophysics, Panjab University, Chandigarh, India; 3 Department of Clinical Hematology and Medical Oncology, Postgraduate Institute of Medical Education and Research, Chandigarh, India; 4 Department of Cytology and Gynaecological Pathology, Postgraduate Institute of Medical Education and Research, Chandigarh, India

**Keywords:** Skin fibroblast culture, Myeloid malignancies, Germline predisposition, Punch biopsy, Germline DNA isolation

## Abstract

Inherited germline variants are now recognized as important contributors to hematologic myeloid malignancies, but their reliable detection depends on obtaining uncontaminated germline DNA. In solid tumors, peripheral blood remains free of tumor cells and therefore serves as a standard source for germline testing. In contrast, peripheral blood often contains neoplastic or clonally mutated cells in hematologic malignancies, making it impossible to distinguish somatic from germline variants. This unique challenge necessitates using an alternative, non-hematopoietic tissue source for accurate germline assessment in patients with hematologic myeloid malignancies. Cultured skin fibroblasts derived from punch biopsies have long been considered the gold standard for this purpose. Nevertheless, most existing protocols are optimized for research settings and lack detailed, patient-centric workflows for routine clinical use. Addressing this translational gap, we present a robust, enzyme-free protocol for culturing dermal fibroblasts from skin punch biopsies collected at the bedside during routine bone marrow procedures. The method details practical bedside collection, sterile transport, mechanical dissection without enzymatic digestion, plating strategy, culture expansion, and high-yield DNA isolation with validated purity. By integrating this standardized approach into routine hematopathology workflows, the protocol ensures reliable germline material with minimal patient discomfort and a turnaround time suitable for clinical diagnostics.

Key features

• This protocol integrates bedside skin punch biopsy with routine bone marrow sampling to minimize patient discomfort and avoid additional invasive procedures.

• It uses an optimized enzyme-free mechanical dissection method with fat removal, fine mincing, and scratched well plating to reduce contamination and improve fibroblast yield.

• It provides an easy-to-follow workflow for primary fibroblast culture, plating, expansion, and harvest, suitable for routine hematopathology laboratories.

• It consistently yields high-quality germline DNA free of hematopoietic contamination, ideal for genetic testing in myeloid malignancies.

## Graphical overview


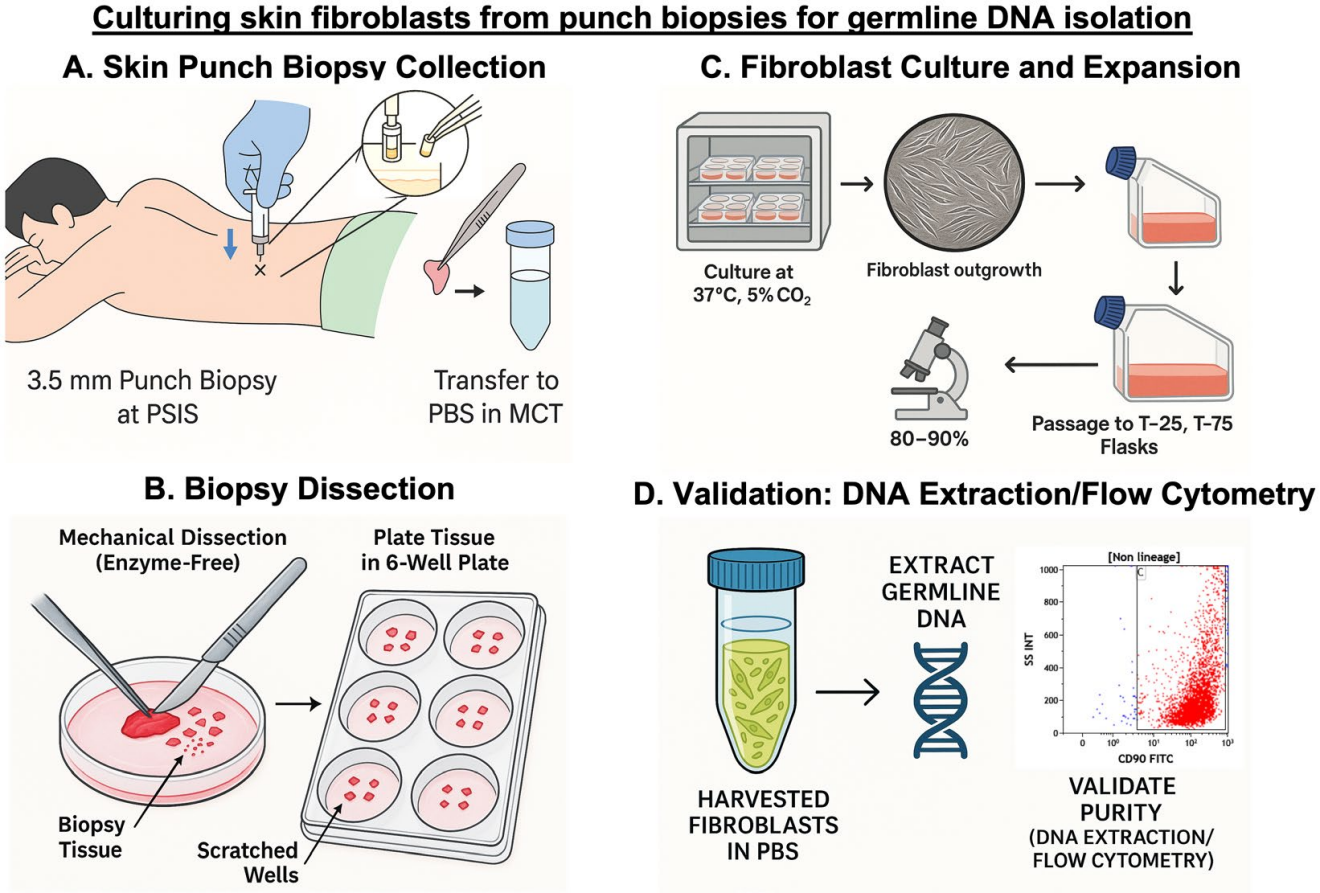


## Background

Myeloid malignancies are clonal disorders of hematopoietic progenitor cells characterized by impaired differentiation and deregulated proliferation. These include acute myeloid leukemia (AML), myelodysplastic neoplasms (MDS), myeloproliferative neoplasms (MPN), and overlapping entities such as MDS/MPN [1]. Traditionally, their pathogenesis has been attributed to somatic mutations that perturb essential processes like self-renewal, proliferation, and differentiation [2]. These mutations can be broadly classified into five categories based on function: signal transduction proteins (e.g., *JAK2, MPL, CALR, FLT3*, and *KIT*), transcription factors (e.g., *CEBPA, RUNX1*, and *ETV6*), epigenetic modifiers (e.g., *TET2, DNMT3A, ASXL1*, and *IDH1/2*), tumor suppressors (e.g., *TP53, WT1*, and *PHF6*), and splicing factors (e.g., *SF3B1, SRSF2*, and *U2AF1*) [3,4]. In recent years, however, there has been increasing recognition that a subset of myeloid neoplasms may arise in the setting of germline predisposition [5–7]. The advent of next-generation sequencing (NGS) has uncovered a growing number of germline variants associated with inherited leukemia predisposition syndromes [8]. The fifth edition of the World Health Organization (WHO) classification of hematolymphoid tumors categorizes such myeloid neoplasms into three groups: (i) those without a pre-existing disorder, including germline mutations in *CEBPA* and *DDX41*; (ii) those with pre-existing thrombocytopenia, involving *RUNX1, ANKRD26*, and *ETV6* mutations; and (iii) those with additional organ dysfunction, such as *GATA2* deficiency or syndromic disorders like Fanconi anemia, telomere biology disorders, Noonan syndrome, and Down syndrome [1].

Compared to the well-established role of germline variants in solid tumors like breast cancer, our understanding of inherited predisposition in hematologic malignancies remains limited [9]. A unique challenge arises from the fact that several genes implicated in myeloid neoplasms, such as *RUNX1, CEBPA*, and *TP53*, can harbor both somatic and germline variants [10,11]. As a result, determining the origin of a detected variant is not always straightforward. In solid tumors, this issue is commonly addressed by paired sampling—comparing the mutation in tumor tissue to that in a non-malignant reference tissue like peripheral blood [12,13]. However, in leukemia and related disorders, both peripheral blood and bone marrow are often involved in the disease process or affected by clonal hematopoiesis, making them unreliable for germline analysis. Consequently, accurate classification of such variants in hematologic malignancies necessitates using alternate, non-hematopoietic tissues.

Among the available non-hematopoietic tissues, cultured skin fibroblasts from punch biopsy specimens have emerged as the most reliable source for germline DNA in patients with hematologic malignancies [14,15]. They offer several advantages, including elimination of contaminating hematopoietic cells, high DNA yield, and reproducibility, making them superior to alternatives such as hair follicles, nail clippings, or buccal mucosa, which often yield inadequate concentration of DNA for clinical-grade assays [14].

While several protocols for culturing fibroblasts from skin biopsies have been published, the majority remain research-oriented and lack direct clinical applicability [16–18]. These protocols often focus on enzymatic digestion techniques or specialized culture conditions, with limited guidance on how they can be effectively integrated into patient care. They also seldom address practical aspects such as bedside sample collection or the need for quick turnaround time in real-world diagnostic settings. To bridge this translational gap, we present a streamlined, enzyme-free protocol specifically optimized for clinical laboratories involved in diagnosing myeloid malignancies. Our method incorporates practical modifications, such as using mechanical separation instead of enzyme-based digestion to separate the epidermis from the dermis, eliminating the need to adhere skin pieces with gelatin, and other simple adjustments that promote faster fibroblast outgrowth. These refinements reduce culture time and contamination risk and are critical when timely and accurate germline results impact patient management. This comprehensive protocol details every step, from bedside collection of the skin punch biopsy alongside routine bone marrow sampling, to standardized fibroblast culture, confluency monitoring, and high-quality DNA extraction for germline variant analysis. The protocol was validated using multiple complementary approaches, including DNA yield assessment, flow cytometry, and immunocytochemistry (ICC). Additionally, the commonly studied *JAK2V617F* single-nucleotide polymorphism—frequently observed in myeloproliferative neoplasms like polycythemia vera, essential thrombocythemia, and primary myelofibrosis—was specifically used as a prototype during validation. Its detection in the patient’s bone marrow DNA and absence in fibroblast-derived DNA confirmed the lack of hematopoietic contamination and reinforced the reliability of the cultured fibroblasts as a source of pure germline DNA.

By emphasizing feasibility, reproducibility, and clinical relevance, our approach provides a practical framework for integrating fibroblast culture into routine diagnostic workflows for assessing hereditary predisposition in myeloid neoplasms.

## Materials and reagents

**Biological materials**

Human skin punch biopsies were collected from the posterior superior iliac spine of patients diagnosed with various hematological malignancies (n = 18) for protocol validation (Table 1). For standardization, skin biopsies from five patients with non-malignant hematological disorders were used to optimize the method. Biopsies were collected at the same time as routine bone marrow aspiration and trephine biopsy procedures. Written informed consent was obtained from all participants, and the Institutional Ethics Committee approved the study.

**Table 1.** Clinical details of patients included for protocol standardization and validation

Patient group	Number of patients	Diagnosis
Standardization set	5	Aplastic anemia (3), immune thrombocytopenia (ITP) (2)
Validation set	18	Acute myeloid leukemia (AML) (12), myelodysplastic syndrome (MDS) (6)

**Reagents**

1. Fetal bovine serum (FBS), heat-inactivated (Gibco, catalog number: 10437028); store at -20 °C for up to 5 years

2. Penicillin-streptomycin (10,000 U/mL) (Gibco, catalog number: 15140122); store at -20 °C for up to 1 year

3. Phosphate buffered saline (PBS), pH 7.2 (Sigma-Aldrich, catalog number: P2272); store at room temperature

4. Dulbecco's modified Eagle medium (DMEM) (Gibco, catalog number: 11995065); store at 4 °C for up to 24 months

5. Trypsin-EDTA (0.25%), phenol red (Gibco, catalog number: 25200056); store at 4 °C for up to 24 months

6. Povidone-iodine solution (10%) (Win Medicare, Betadine 10% solution); store below 22–30 °C, protected from light and moisture

7. Injection lignocaine hydrochloride (2%) (Zydus Healthcare Ltd., Xylocaine 2%); store below 22–30 °C, protected from light and moisture

**Solutions**

1. 10% complete DMEM (see Recipes)

**Recipes**

**1. 10% complete DMEM**

**Table:** 

Reagent	Final concentration	Volume
DMEM	1×	45 mL
FBS	10%	5 mL
Penicillin-Streptomycin	1%	0.5 mL

This should be freshly prepared when required. Before preparation, filter the required volume of DMEM through a 0.2 μm syringe filter and the required volume of FBS through a 0.45 μm syringe filter. After filtering, combine as per the recipe and aliquot into a 50 mL graduated centrifuge sterile tube. Store the freshly prepared complete DMEM at 4 °C.

**Laboratory supplies**

1. Pipette, 10 μL (RAININ, catalog number: 17014388)

2. Pipette, 200 μL (Sartorius, catalog number: 16598143)

3. Pipette, 1,000 μL (Eppendorf, catalog number: P37952F)

4. Pipette tips, 10 μL (Axygen, catalog number: T-300)

5. Pipette tips, 200 μL (Tarsons, catalog number: 52101Y)

6. Pipette tips, 1,000 μL (Tarsons, catalog number: 521020X-B)

7. Graduated centrifuge tubes, 15 mL (Tarsons, catalog number: 546021)

8. Graduated centrifuge tubes, 50 mL (Tarsons, catalog number: 546041)

9. Gamma-irradiated sterile Petri dish, 90 mm (Abdos Life Sciences, catalog number: P10910)

10. Graduated microcentrifuge tubes, 1.5 mL (Axygen, catalog number: MCT-150-C-S)

11. Graduated microcentrifuge tubes, 2.0 mL (Axygen, catalog number: MCT-200-C-S)

12. Cell culture plate, 6-well, tissue culture treated (Corning, catalog number: 3516)

13. Tissue culture flask, T-75 (Thermo Scientific, catalog number: 156499)

14. Tissue culture flask, 25 cm^2^, canted neck, 50 mL (Falcon, catalog number: 353108)

15. Sterile single-use hypodermic syringe, 5 mL (DISPO VAN)

16. Sterile single-use hypodermic syringe, 20 mL (DISPO VAN)

17. Sterile single-use needle, 23G × 1” (Safeway)

18. Disposable nitrile gloves (VWR, catalog number: 1122371)

19. Kimwipes (KIMTECH, catalog number: 34155)

20. Syringe filter, 0.45 μm (PALL Life Sciences, catalog number: PN 4614)

21. Syringe filter, 0.2 μm (PALL Life Sciences, catalog number: PN 4612)

22. Parafilm (BEMIS, catalog number: PM-996)

23. Centrifuge tube rack (Falcon or any compatible rack)

24. Sterile gauze (from local vendor)

25. Sterile cotton (from local vendor)

*Note: All items that come into contact with biopsy material or cell cultures must be sterile, whether supplied as pre-sterilized single-use consumables or sterilized in-house before use.*

## Equipment

1. CO_2_ cell culture incubator (NUAIRE, model: NU-5710E)

2. Biosafety cabinet, Class II Type A2 (ESCO Life Sciences, model: Airstream)

3. Centrifuge, with swing bucket rotor (REMI, model: CM-8 PLUS)

4. Inverted microscope (RADICAL, model: RTC-7)

5. Sterile biopsy punch 3.5 mm diameter, single use (Paramount, catalog number: BPS3.5)

6. Sterile surgical blades No. 22 (GLASS VAN, catalog number: 9512442321)

7. Scalpel handle No. 4, 13.5 cm (GDC, catalog number: 10-100-04E)

8. Straight dissecting forceps, 11.5 cm (from local vendor)

9. Straight pointed scissors, 5" (from local vendor)

10. Sponge holder forceps, 178 mm (from local vendor)

11. Draping sheet (from local vendor)

12. UV light sterilizer bag (from local vendor)

## Procedure

**A. Pre-biopsy preparation and transport setup**

1. Begin preparation one day before the planned procedure.

2. Label sterile 1.5 or 2 mL microcentrifuge tubes (MCTs) for biopsy collection. Add 1 mL of sterile PBS to the MCT, seal securely with parafilm, and store at 4 °C.

3. Autoclave the complete surgical set, including forceps, scissors, sterile gauze, sterile cotton, and draping sheet.

4. On the day of the procedure, place the PBS-filled MCTs, a 3.5 mm biopsy punch, and the sterilized surgical set into a UV-light sterilizer bag. Keep the bag secured until used and transport it directly to the procedure room.

**B. Skin biopsy collection procedure**

**Figure 1.**
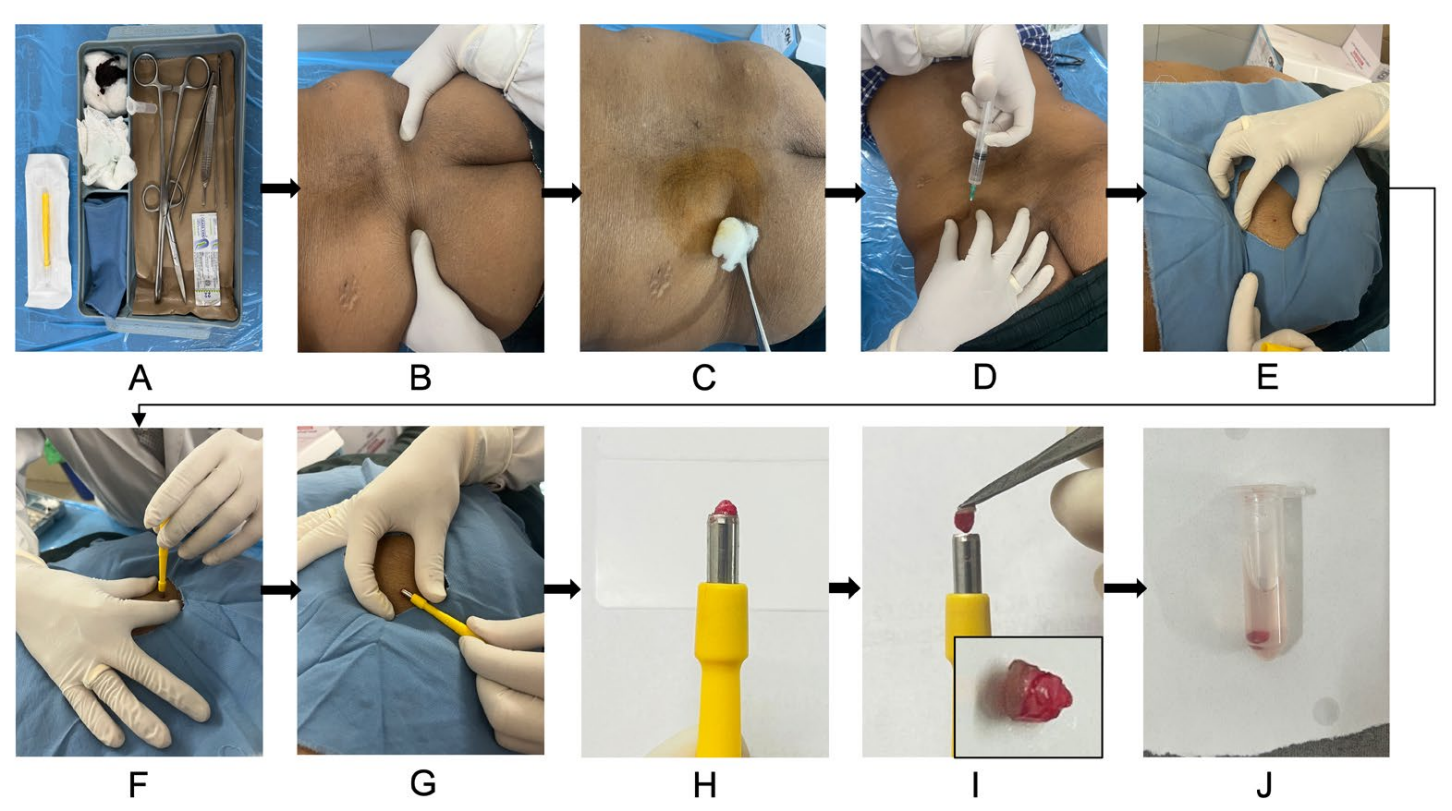
Skin punch biopsy collection for dermal fibroblast culture. (A) Sterile biopsy kit setup with all required instruments and collection tubes. (B) Patient is positioned prone, and the posterior superior iliac spine (PSIS) is located for sampling. (C) Skin cleaned thoroughly with spirit swab and povidone-iodine solution. (D) Local anesthesia infiltration with 2% lignocaine using a sterile syringe and needle. (E) Sterile draping is placed over the biopsy site. Stretch the skin to ensure proper wound approximation after biopsy. (F) Performing a punch biopsy with gentle downward rotation. (G–I) Lifting out the biopsy core from the site and picking out with the toothed forceps (inset in panel I shows the skin punch biopsy). (J) Transferring the biopsy into a sterile microcentrifuge tube containing PBS.

Skin punch biopsy for fibroblast culture is performed during the same sitting as the scheduled bone marrow aspiration/biopsy. Using the same site—the posterior superior iliac spine (PSIS) region—allows both samples to be collected with a single local anesthetic infiltration, minimizing additional pain for the patient and improving procedural efficiency (Figure 1).

1. Label the MCT when setting up the tray before the procedure, with the patient's name/ID and collection date to ensure traceability.

2. Instruct the patient to lie in the prone position on a procedure table or bed covered with a sterile drape or sterile sheet. Wear sterile disposable gloves. Palpate and mark the PSIS, which serves as the standard site for both the skin punch and bone marrow procedures.

3. Thoroughly disinfect the PSIS region using a sterile spirit swab, then apply 10% povidone-iodine solution, allowing it to dry and act for 5 min. Repeat with a fresh spirit swab to remove excess solution and re-sterilize the area.

4. Drape the biopsy site with a sterile drape cloth, ensuring the surrounding area remains covered.

5. Load a sterile 5 mL syringe fitted with a 23-gauge needle and draw up 7 mL of 2% lignocaine hydrochloride injection. Infiltrate 2 mL subcutaneously into the marked area to anesthetize an area approximately 1 cm in diameter for the skin punch. Infiltrate the remaining 5 mL into the deeper soft tissue and periosteum to anesthetize the bone for the subsequent bone marrow procedure as outlined in standard protocols [19]. Allow 5 min for the anesthetic agent to take full effect.

6. Remove the sterile 3.5 mm disposable biopsy punch from its packaging. Stretch the skin at the marked site between the fingers. This is important because, once the punch biopsy is taken, the skin naturally retracts to close the defect into a narrow elliptical slit, helping the wound edges approximate on their own without the need for sutures.

7. Position the punch perpendicular to the stretched skin surface directly over the anesthetized site. Apply gentle, steady downward pressure while rotating the punch in a clockwise motion. Full depth is reached when the punch advances through the dermis, and resistance suddenly decreases.

8. Once the full depth is reached, tilt the punch 90° so that it becomes parallel to the skin surface. This maneuver helps to lift and separate the tissue core cleanly so that the specimen pops out into the hollow cylinder of the punch.

9. If the core remains attached, gently grasp and lift it with sterile toothed forceps. Sterile scissors can be used to snip any remaining dermal attachments if needed.

10. Open the pre-prepared MCT containing cold sterile PBS. Using sterile toothed forceps, carefully remove the skin specimen from the biopsy punch and transfer it directly into the tube. Close the cap securely and gently invert or shake the tube to ensure the tissue is fully suspended in the PBS.

11. If minor bleeding occurs at the biopsy site, apply gentle pressure with sterile Gauze or cotton until hemostasis is achieved. Proceed with the bone marrow procedure per standard practice at the same site.

12. Return the sealed MCT to the UV-sterilized transport box.

13. Immediately transport the sealed box to the cell culture laboratory and initiate fibroblast isolation and culture within 1 h of collection.

*Note: Combining the skin punch biopsy with the bone marrow procedure saves the patient additional discomfort, reduces anesthesia volume, and improves efficiency.*

**Caution:** Minimize the time from biopsy collection to processing to preserve fibroblast viability and prevent microbial contamination.

**C. Punch biopsy dissection and plating**

**Figure 2.**
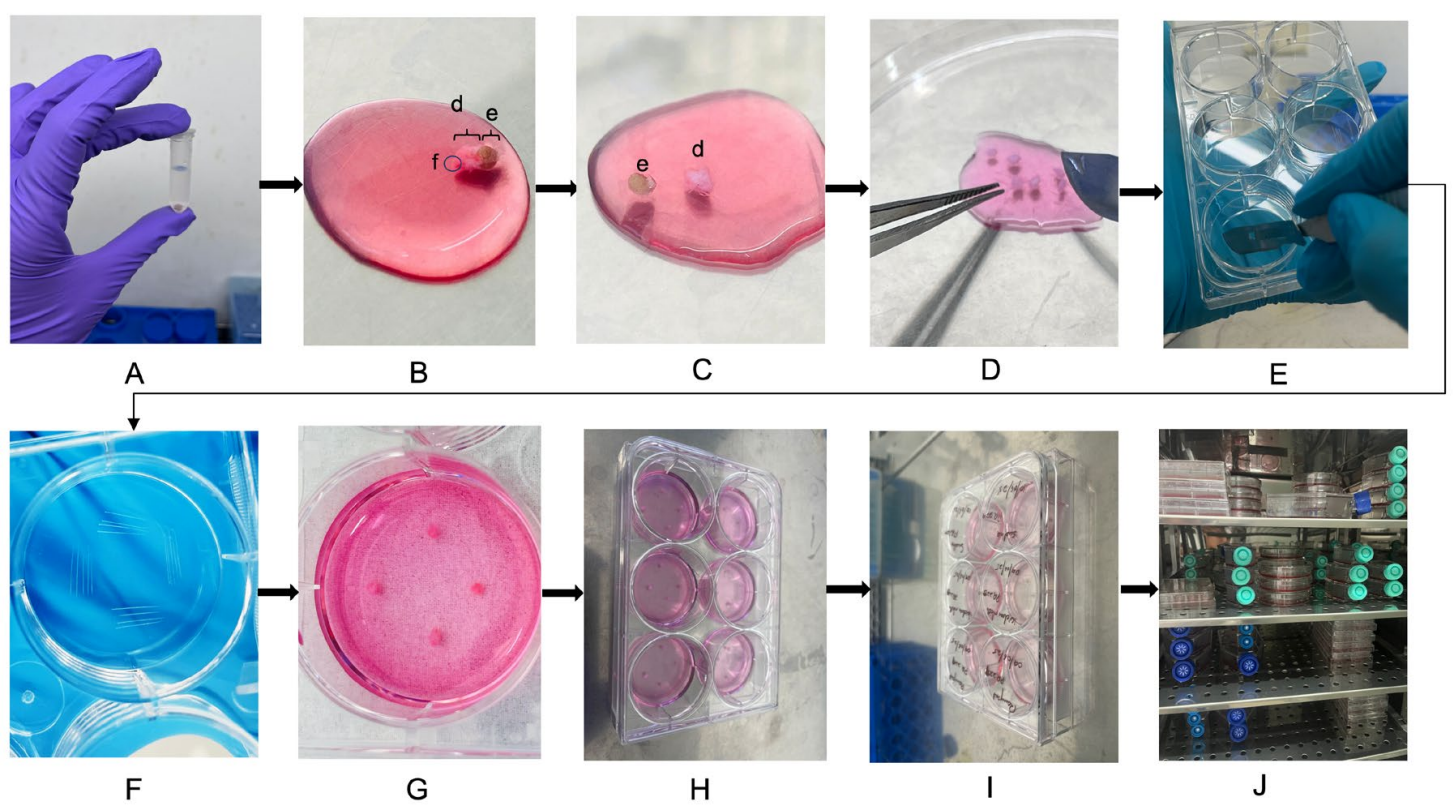
Punch biopsy dissection and plating for fibroblast culture. (A) Washing the skin biopsy tissue 3–4 times with sterile 1× PBS to remove residual blood and debris. (B) Placing washed tissue in a sterile Petri dish containing freshly prepared 10% complete DMEM. The epidermis, e; dermis, d; and subcutaneous fat, f, are labeled. (C–D) Mechanically dissecting the biopsy using sterile forceps and a scalpel to separate epidermis, trim excess fat (if any), and chop the dermal piece into small, evenly sized fragments (~0.3–0.5 mm). (E–F) Scratching the base of each well with a sterile scalpel to promote tissue attachment. (G–H) Placing four tissue fragments evenly along the scratched areas in each well. (I) Labeling the 6-well tissue culture plate with patient ID and date. (J) Culturing the plate inside the humidified CO_2_ incubator for fibroblast outgrowth.

1. Wear sterile disposable gloves throughout.

2. Wipe down all aliquots of media (in 50 mL graduated centrifuge sterile tube), PBS bottles, Petri dishes, 6-well plates, forceps, scalpel handles, and blades with 70% ethanol before placing them inside the biosafety cabinet. UV-sterilize the cabinet for 30 min. After UV exposure, clean the work surface and pipettes again with 70% ethanol.

3. Remove the MCT containing the skin biopsy from the transport box. Wash the tissue 3–4 times with sterile 1× PBS by pipetting up and down around the tissue fragment to gently remove any residual blood or debris (Figure 2). Take care not to aspirate the biopsy into the pipette tip.

4. Add 500 μL of freshly prepared 10% complete DMEM to a sterile 90 mm Petri dish. Transfer the washed tissue directly into the dish to remain immersed during dissection.

5. Hold the tissue steady with sterile forceps. Using a sterile scalpel blade, make clean, smooth cuts to separate the epidermis from the dermis and remove excess subcutaneous fat. Avoid ragged edges, which can hinder cell outgrowth.

6. Once the epidermis is removed, chop the remaining dermal piece into 6–8 evenly sized fragments, each approximately 0.3–0.5 mm in size. Smaller, uniformly sized pieces promote faster and more consistent fibroblast migration.

*Note: This protocol uses an enzyme-free mechanical dissection approach to minimize processing time and reduce contamination risk.*

7. Label a sterile 6-well tissue culture plate clearly with the patient’s ID and date of procedure.

8. Using a sterile scalpel blade, gently scratch four small lines in the base of each well to help the tissue fragments adhere.

9. Place four biopsy pieces evenly along the scratched areas in one well, ensuring the pieces do not touch each other.

10. Allow the tissue pieces to settle for 5 min to facilitate initial attachment. Then, gently add 800 μL to 1 mL of fresh 10% complete DMEM to each well to cover the tissue fully. Close the 6-well plate with its sterile lid.

11. After plating, clean the working area, forceps, and scalpel thoroughly with 70% ethanol. Return any remaining media and PBS to storage at 4 °C.

**D. Culture conditions and maintenance**

**Figure 3.**
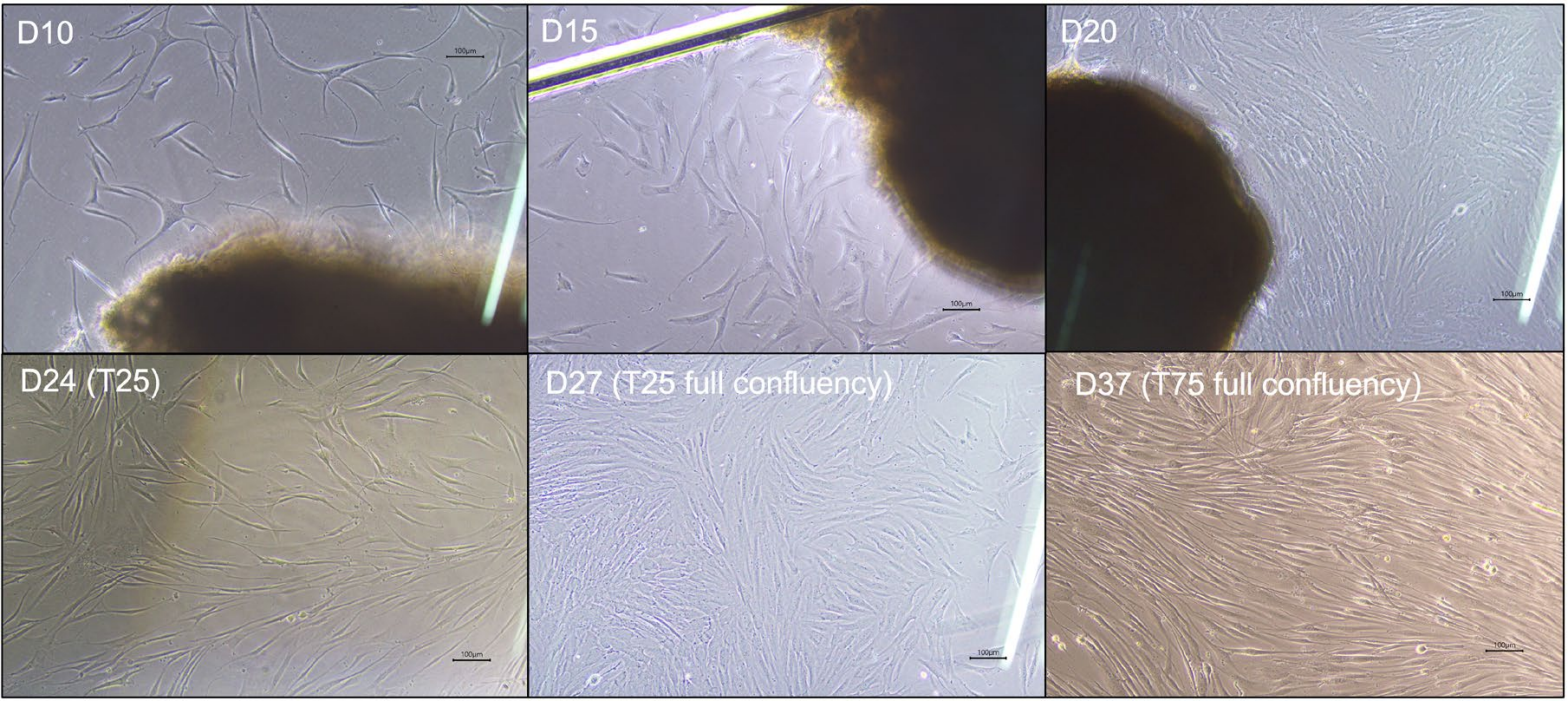
Morphological progression of fibroblast outgrowth and expansion during culture and passaging. Phase-contrast images showing representative fibroblast migration and proliferation from initial tissue plating through expansion stages. **D10–D15:** Early fibroblast outgrowth emerges as elongated spindle-shaped cells extending from biopsy fragments in the 6-well culture plate. **D20:** Cells reach approximately 80%–90% confluency in the 6-well plate. **D24 (T25):** Cells continue to proliferate after first passage into a T-25 flask. **D27 (T25 full confluency):** Fibroblasts reach 80%–90% confluency in T-25 flask. **D37 (T75 full confluency):** Expanded fibroblast culture in T-75 flask showing dense, confluent monolayer suitable for high-yield DNA isolation. Scale bars, 100 µm.

1. Place the 6-well plate in a humidified CO_2_ incubator set at 37 °C with 5% CO_2_.

2. Check the cultures daily under an inverted microscope to ensure that tissue pieces remain fully submerged and to monitor for any signs of contamination.

3. On day 2, gently add 200 μL of fresh 10% complete DMEM to each well. Continue to add 200 μL of fresh medium every alternate day to maintain nutrient levels.

4. By day 10, observe fibroblast outgrowth under the inverted microscope; look for elongated spindle-shaped cells migrating from the tissue fragments (Figure 3).

5. Once visible cell outgrowth is established, change the medium every second day to maintain optimal culture conditions.

6. When fibroblasts outgrow the well surface and reach approximately 80%–90% confluency, prepare for passaging to expand the culture.

7. To ensure full traceability and quality assurance, maintain a detailed culture log for each patient, including patient ID, procedure date, confluency status, medium change dates, and passage details.

**Critical:** Always thaw all media, PBS, and trypsin-EDTA at room temperature for at least 1h before use to ensure consistent cell viability and detachment efficiency.

**E. Passaging and expansion**

1. Prepare the biosafety cabinet: run UV sterilization for 30 min and then wipe down the work surface and all equipment with 70% ethanol.

2. Carefully aspirate and discard the spent medium from the well. Add 1 mL of 0.25% Trypsin-EDTA to detach the adherent fibroblasts. Incubate the plate at 37 °C in the CO_2_ incubator for 10 min.

3. After incubation, add 1 mL of 10% complete DMEM to neutralize the trypsin. Gently pipette up and down to fully detach any remaining cells.

4. Transfer the entire cell suspension and the original biopsy tissue pieces into a sterile 15 mL centrifuge tube.

*Note: Including the seeded biopsy pieces during passaging can enhance downstream yield by allowing any remaining fibroblasts to continue proliferating.*

5. Centrifuge at 400× *g* for 5 min. Carefully discard the supernatant. Resuspend the cell pellet in 500 μL of fresh 10% complete DMEM.

6. Meanwhile, add 5 mL of fresh 10% complete DMEM to a sterile T-25 flask.

7. Transfer the resuspended cells into the T-25 flask and gently rock the flask side to side to distribute the cells evenly. Label the flask as Passage 1, including the patient ID and date.

8. Incubate the flask at 37 °C with 5% CO_2_. Monitor cell attachment and morphology every alternate day. Replace the medium every 4–5 days.

9. Once fibroblasts reach 80%–90% confluency in the T-25 flask, repeat the trypsinization, neutralization, and centrifugation steps (E1–5) as described above (Figure 3).

10. Add 8 mL of fresh 10% complete DMEM to a sterile T-75 flask. Resuspend the new pellet in 1 mL of medium and transfer to the T-75 flask. Gently rock to evenly distribute the cells (Passage 2).

*Note: At Passage 2, residual biopsy tissue pieces are discarded, as sufficient fibroblast expansion has been achieved.*

11. Incubate and monitor as before, changing the medium every 5 days.

12. Once the T-75 flask reaches 80%–90% confluency, harvest the cells by trypsinization and centrifugation, discard the supernatant, and resuspend the final pellet in 200 μL of PBS for downstream germline DNA extraction.

## Validation of protocol

**Figure 4.**
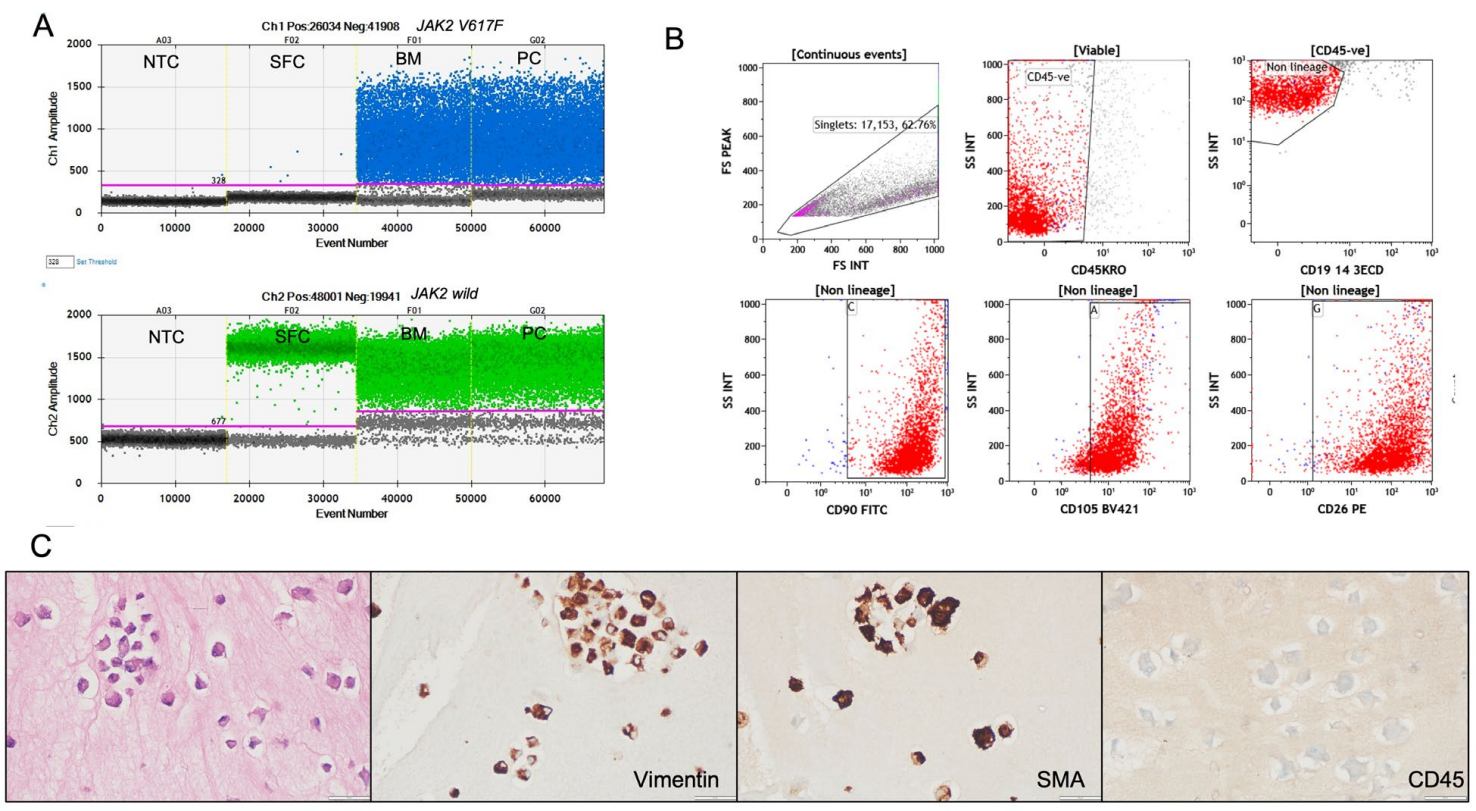
Validation of cultured dermal fibroblasts for germline DNA analysis. (A) Representative droplet digital PCR (ddPCR) plots for *JAK2* V617F mutation detection. The *JAK2* V617F mutant allele is labeled with FAM (blue) and the wild-type allele with HEX (green). The mutation is detected in bone marrow aspirate (BM) and positive control (PC) DNA but absent in DNA extracted from cultured skin fibroblasts (SFC) from the same patient, confirming the purity of the fibroblast-derived germline DNA. NTC: no template control. (B) Flow cytometry characterization of cultured fibroblasts showing strong expression of fibroblast markers CD90, CD105, and CD26, with no expression of hematopoietic lineage markers (CD45, CD19, CD3, CD14), supporting fibroblast identity and absence of hematopoietic cell contamination. (C) Immunocytochemistry (ICC) on cell blocks prepared from cultured fibroblasts, demonstrating strong cytoplasmic staining for vimentin and smooth muscle actin (SMA) and negative staining for CD45, confirming mesenchymal fibroblast phenotype and non-hematopoietic origin. Scale bars, 50 µm.

This protocol was standardized using skin punch biopsies from five patients with non-malignant hematological disorders and validated with an additional 18 patients with hematological malignancies by the following complementary approaches:

1. DNA extracted from the fibroblasts grown to confluency in T-75 flasks (for all 18 cases) showed a mean concentration of 252.38 ng/μL, ranging from 53.9 to 822.5 ng/μL, demonstrating high and consistent yield for downstream analysis (Table S1).

2. To confirm the adequacy of dermal fibroblasts as a representative source of germline DNA, the same was extracted and tested for the *JAK2V617F* variant. For this, a culture from a patient diagnosed with polycythemia vera (known to harbor the *JAK2V617F* mutation) was used as a positive reference. The mutation was detected in the patient's bone marrow sample. However, it was negative in the DNA extracted from the cultured dermal fibroblasts, confirming the absence of this common somatic mutation in the germline tissue and supporting the purity of the fibroblast-derived DNA (Figure 4).

3. Flow cytometry characterization was performed on fibroblasts cultured from six patient samples. Approximately 1 × 10^3^ to 2 × 10^3^ cells were harvested, stained, and analyzed for each sample. The cultured dermal fibroblasts in all these cases were strongly positive for fibroblast markers CD90, CD105, and CD26, confirming their mesenchymal lineage identity. Notably, the cells were negative for hematopoietic markers such as CD45, CD19, CD3, and CD14, confirming the absence of contaminating hematopoietic cells (Figure 4, Table S2).

4. ICC was performed on cell blocks prepared from cultured fibroblasts derived from two representative patient samples, as an additional validation step to confirm cell identity and purity. For cell block preparation, the harvested fibroblast cell suspension was first fixed in 10% neutral buffered formalin and processed using standard cytopathology protocols to create paraffin-embedded cell blocks, similar to techniques routinely applied for diagnostic cytology samples. This approach preserves cellular morphology and allows for reliable immunostaining. Immunostaining was then carried out on serial sections using antibodies against vimentin, smooth muscle actin (SMA), and the hematopoietic marker CD45. The cultured fibroblasts demonstrated strong cytoplasmic positivity for vimentin and SMA, both of which are consistent with a mesenchymal fibroblast phenotype. No staining was observed for CD45, confirming the absence of hematopoietic contamination (Figure 4, Table S3). These ICC results further validate that the cultured cells were authentic dermal fibroblasts with a non-hematopoietic origin, supporting the protocol's suitability for generating pure germline DNA samples.

## General notes and troubleshooting

**General notes**

1. Always perform all pre-biopsy preparation steps, tissue handling, and cell culture work in a certified Class II biosafety cabinet using strict aseptic technique.

2. Use only sterile, pre-filtered solutions and equipment; store all reagents at the recommended temperatures to maintain sterility and effectiveness.

3. Transport skin biopsies in cold sterile PBS and process them within 1–2 h of collection for optimal fibroblast viability.

4. Small, evenly cut tissue pieces with smooth edges improve fibroblast outgrowth and reduce contamination risk.

5. Patient-to-patient variability in outgrowth can occur; keep detailed logs for each sample to track culture progress.

6. This protocol is optimized for patients with hematologic malignancies but may be adapted for other tissues where germline DNA is needed.

**Troubleshooting**

Problem 1: Little or no fibroblast outgrowth, variability in yield, or absence of cell growth.

Possible causes: Biopsy not plated the same day; tissue processed too late or degraded; pieces too large or ragged; patient-specific clinical or genetic factors.

Solutions: Always process and plate the biopsy within 1–2 h of collection. Dissect tissue into small, evenly sized pieces (~0.3–0.5 mm) with clean edges to promote outgrowth. Adjust medium composition if needed to support growth.

Problem 2: Contamination in culture wells.

Possible causes: Inadequate aseptic technique; nonsterile instruments; contaminated media.

Solutions: Work only in a certified Class II biosafety cabinet. Disinfect surfaces and instruments before and after use. UV-sterilize the workspace and wipe down with 70% ethanol. Use only sterile, pre-filtered media and solutions.

Problem 3: Non-adherence of skin biopsy tissue to the plate.

Possible causes: Tissue pieces too large; residual subcutaneous fat; scratches not appropriately made.

Solutions: Trim tissue to the recommended size (~3 mm × 1 mm) and carefully remove excess fat with a sterile scalpel. Make gentle scratches on the plate surface to help tissue adhere and allow it to settle for 5 minutes before adding medium.

Problem 4: Low DNA yield.

Possible causes: Cells harvested too early; incomplete outgrowth.

Solutions: Wait until cultures reach 80%–90% confluency before harvesting. Include the biopsy tissue during the first passage to boost cell numbers.

Problem 5: Cells detach prematurely.

Possible causes: Medium changes too forcefully; tissue not properly attached.

Solutions: Pipette gently during medium changes. Ensure scratches are adequate and tissue is well-positioned to promote attachment.

## Supplementary information

The following supporting information can be downloaded here:

1. Table S1. DNA yield from cultured dermal fibroblasts (validation cohort, n = 18)

2. Table S2. Flow cytometry characterization of cultured fibroblasts (n = 6)

3. Table S3. Immunocytochemistry (ICC) on fibroblast cell blocks (n = 2)
